# Association between calculated remnant cholesterol levels and incident risks of Alzheimer’s disease among elderly patients with type 2 diabetes: a real-world study

**DOI:** 10.3389/fendo.2024.1505234

**Published:** 2024-11-29

**Authors:** Huimeng Jia, Liuyu Zhang, Huijuan Liao, Yiming Li, Pan Liu, Qin Shi, Bo Jiang, Xian Zhang, Yufeng Jiang, Zhihong Nie, Mei Jiang

**Affiliations:** ^1^ Department of General Medicine, Gongli Hospital of Shanghai Pudong New Area, Shanghai, China; ^2^ Department of Neurology, Haishu District People’s Hospital, Ningbo, China; ^3^ Department of Cardiology, Gongli Hospital of Shanghai Pudong New Area, Shanghai, China; ^4^ Department of Neurology, Gongli Hospital of Shanghai Pudong New Area, Shanghai, China

**Keywords:** Alzheimer’s disease, remnant cholesterol, real world study, cholesterol, biomarker

## Abstract

**Objective:**

Alzheimer’s disease (AD) is a leading cause of dementia, with a rising global burden. Remnant cholesterol (RC), a component of triglyceride-rich lipoproteins, has been implicated in cardiovascular diseases and metabolic disorders, but its role in AD remains unclear. This study investigated the association between RC levels and the risk of AD among elderly patients with type 2 diabetes (T2D) in a real-world clinical setting.

**Methods:**

We conducted a retrospective cohort study using electronic medical records from Gongli Hospital of Shanghai Pudong New Area, covering the period from 2013 to 2023. The study included 15,364 elderly patients aged 65-80 years with T2D. RC levels were calculated using the equation. The primary outcome was the diagnosis of AD, validated by neurologists using ICD-10-CM code G30. Cox proportional hazards models were employed to estimate hazard ratios (HRs) for AD across quartiles of RC levels, adjusting for potential confounders.

**Results:**

Over a mean follow-up of 3.69 ± 1.33 years, 312 new cases of AD were identified. A U-shaped relationship was observed between RC levels and AD risk, with the lowest risk associated with RC levels between 0.58-0.64 mmol/L. Both lower (<0.52 mmol/L) and higher (≥0.77 mmol/L) RC levels were linked to increased AD risk. Compared to the reference group (Q2: 0.52-0.64 mmol/L), the adjusted HRs (95% CI) for the lowest and highest quartiles were 1.891 (1.368-2.613) and 1.891 (1.363-2.622), respectively. Each 1 mmol/L increase in RC was associated with a 3.47-fold higher risk of AD (HR=4.474, 95% CI 2.330-8.592).

**Conclusion:**

RC levels may serve as a predictive biomarker for AD risk, with both extremes posing a higher risk. Future studies should explore the mechanistic pathways and potential interventions targeting RC to prevent AD in high-risk populations.

## Introduction

Alzheimer’s disease (AD) is an irreversible neurodegenerative disorder and one of the most common neurodegenerative diseases among the elderly. It is a major cause of dementia, accounting for approximately 80% of all dementia diagnoses, with the number of cases projected to rise continuously over the next 30 years (1, 2). Annually, the direct and indirect costs associated with AD are nearly $500 billion (3). Patients with AD experience a decline in memory, which progressively leads to cognitive impairment and ultimately death. The pathogenesis of AD is complex and not fully understood. The widely accepted mechanisms include neuronal and synaptic loss, extracellular deposition of beta-amyloid protein (Aβ), formation of neuritic plaques (NPs), and intracellular hyperphosphorylation of tau protein, leading to neurofibrillary tangles (NFTs) (4). There is a correlation between energy and lipid metabolism and the pathogenesis of AD, with both NPs and NFTs formation closely related to cholesterol metabolism (5–7). This suggests that cholesterol metabolism might play a crucial role in the development of AD. Several clinical studies have indicated an association between hypercholesterolemia and the onset of AD (8–10), but there is a lack of sufficient clinical data on the specific components of cholesterol and their relationship with AD.

Remnant cholesterol (RC), also known as cholesterol in triglyceride-rich lipoproteins (TRLs), includes cholesterol from very low-density lipoprotein and intermediate-density lipoprotein in the fasting state, and cholesterol from chylomicron remnants in the non-fasting state (11). Recent clinical evidence has shown that RC is associated with increased risks of coronary artery disease, diabetic complications, hypertension, and kidney-related diseases (12–15). However, studies on the association between RC and the onset of AD are few. Therefore, we designed this study, based on the real-world data, to analyze the association between RC levels and the incidence of AD.

## Methods

### Study design and population

The Gongli Hospital of Shanghai Pudong New Area was established in 1943. The hospital has 1000 beds and services 800,000 residents from the surrounding communities, mainly for chronic diseases of the elderly. It had nearly two million outpatients and emergency patients annually. The Department of General Medicine was founded in 2014 and has 1,000 inpatients and 50,000 outpatients, including 20,000 outpatients with diabetes and 1,000 outpatients with AD. Meanwhile, the General Medicine Department provides clinical diagnosis and treatment, physician training, scientific research, and other capacity improvement services for 8 community healthcare centers. It also assists them with public health services for about 250,000 elderly people, including anthropometry, demographics, laboratory data, imaging, diagnosis, medicine use condition, health data sharing, and conducting follow-ups, disease monitoring and interventions.

We retrieved data from the electronic medical records (EMRs) in Gongli Hospital from 2013 to 2023. Patients with type 2 diabetes were identified by using the SUPREME-DM criteria (16, 17), which was described as the following: a) 1 or more of the International Classification of Disease, Tenth Revision, Clinical Modification (ICD-10-CM) codes for type 2 diabetes associated with in-patient encounters; b) 2 or more ICD-10-CM codes associated with outpatient encounters on different days within 2 years; c) combination of 2 or more of the following associated with outpatient encounters on different days within 2 years: 1) ICD-10-CM codes associated with outpatient encounters; 2) fasting glucose level ≥ 7.0 mmol/l; 3) 2-hour glucose level ≥ 11.1 mmol/l; 4) random glucose ≥ 11.1 mmol/l; 5) HbA1c ≥ 6.5%; and 6) prescription for an antidiabetic medication. A total of 42,058 patients were identified in the analytic dataset. After additional exclusion criteria applied including patients with age < 65 years old or >80 years old (n=25,645), incomplete information (n=174), and patients with a history of AD at the first hospital visit (n=875), the finalized analytic dataset compromised 15,364 individuals with 6,708 male and 8,656 female. The study protocol and the analytic plan was approved by the Institutional Review Boards of Gongli Hospital (KY-2024-07-26). Informed consent was not obtained from individual patients because we used de-identified data from EMRs.

### Exposures and covariates of interest in this study

As RC could not be directly measured in Gongli Hospital, we used calculated RC levels as the exposure. The equation RC = TC - HDL - LDL was used to determine the RC levels (18, 19). Other patient information, including age, sex, height, weight, diabetes duration, blood pressure, family history, smoking, alcohol consumptions, lab measurements and use of medications were extracted from the EMRs. All the lab profile measurements were performed using centralized methods in the laboratories of Gongli Hospital. The body mass index (BMI) was calculated using the equation weight (kg) divided by the square of height (m^2^). The estimated Glomerular Filtration Rate (eGFR) was calculated using the Chronic Kidney Disease Epidemiology Collaboration (CKD-EPI) equation (20).

### The primary outcome

The primary outcome in this study was the diagnosis of AD by using the ICD-10-CM code G30. We further validated the diagnosis by a group of neurologists blinded to the study by chart review. The accuracy of the diagnosis was 97.1% with a sensitivity of 98.1% and a specificity of 99.9%. The person-years were calculated from the date of the first hospital visit at Gongli Hospital to the date of diagnosis of the outcome, death of inpatients or December 31, 2023.

### Statistical analysis

Data were presented as follows: normally distributed variables as mean ± standard deviation, skewed variables as median with interquartile ranges, and categorical variables as frequencies and percentages. For intergroup comparisons, Student’s t-tests were applied to normally distributed variables, Wilcoxon rank-sum tests to skewed variables, and Chi-square tests to categorical variables. Cox proportional hazards regression models estimated hazard ratios (HRs) for the primary outcome across different categories of RC quartiles and as a continuous variable. Dummy variables represented categories of RC levels, and the linearity of RC categories was evaluated by assigning ordinal numeric values to each category within the model. The proportional hazards assumption was verified using graphical methods and by including time-by-covariate interactions in the models, which upheld the proportionality assumptions. Adjustments were made in all analyses for age, sex, duration of diabetes, family history of diabetes, BMI, systolic blood pressure, HbA1c, LDL cholesterol, HDL cholesterol, triglycerides (TG), C-reactive protein (CRP), estimated glomerular filtration rate (eGFR), smoking status, diabetic complications, and the use of antihypertensive, glucose-lowering, lipid-lowering, and antiplatelet or anticoagulant drugs. Stratified analyses considered different patient demographics and clinical backgrounds, such as age, sex, BMI, HbA1c levels, eGFR, smoking status, and baseline medications. Statistical significance was set at P < 0.05. All analyses were executed using R software version 4.3.0 (R Foundation for Statistical Computing, Vienna, Austria).

## Results

### Baseline characteristics

A total of 15,364 participants were included in this study, comprising 6,708 males and 8,656 females, with males accounting for 43.66%. The average age of participants was 74.1 ± 6.8 years, and the average follow-up period was 3.69 ± 1.33 years. The overall baseline RC level was 0.64 (0.52-0.77) mmol/L. Participants were divided into quartiles based on RC levels: Q1: < 0.52 mmol/L (n=4006), Q2: 0.52-0.64 mmol/L (n=3875), Q3: 0.64-0.77 mmol/L (n=3659), Q4: ≥ 0.77 mmol/L (n=3824). The baseline data for each group are shown in **Table 1**.

**Table 1:** Baseline characteristics of patients according to different residual cholesterol levels.

	Baseline residual cholesterol (mmol/L)			
	Quartile 1	Quartile 2	Quartile 3	Quartile 4
Participants (n)	4006	3875	3659	3824
Age (years)	74.4 ± 6.9	74.1 ± 6.7	74.0 ± 6.7	73.9 ± 6.7
Male (%)	44.0	43.6	43.1	43.7
Body mass index (kg/m^2^)	23.5 (20.7-27.7)	23.5 (20.9-27.8)	23.6 (20.9-27.5)	23.4 (20.8-27.3)
Blood pressure (mmHg)				
Systolic	134.6 ± 12.2	134.6 ± 12.4	134.8 ± 12.1	134.5 ± 11.5
Diastolic	73.3 ± 7.0	73.4 ± 6.9	73.4 ± 6.7	73.4 ± 6.4
Total cholesterol (mmol/L)	3.93 (3.41-4.53)	4.10 (3.56-4.71)	4.22 (3.71-4.82)	4.32 (3.79-4.94)
Low-density lipoprotein cholesterol (mmol/L)	2.25 (1.83-2.76)	2.35 (1.90-2.88)	2.39 (1.96-2.90)	2.39 (1.93-2.94)
High-density lipoprotein cholesterol (mmol/L)	1.17 (0.99-1.38)	1.12 (0.96-1.31)	1.09 (0.93-1.27)	1.03 (0.88-1.20)
Triglycerides (mmol/L)	1.02 (0.95-1.09)	1.27 (1.21-1.34)	1.54 (1.47-1.61)	1.89 (1.79-2.02)
HbA1c (%)	6.7 (6.2-7.5)	6.8 (6.3-7.6)	6.8 (6.3-7.6)	7.0 (6.4-7.9)
Estimated GFR (mL/min/1.73 m^2^) (%)				
< 60	21.3	20.5	21.0	21.3
60–89	38.0	40.0	40.3	40.5
≥ 90	40.7	39.5	38.7	38.2
Current smokers (%)	19.0	18.5	17.8	19.5
Use of medications (%)				
Glucose-lowering	69.3	72.9	76.6	73.3
Antihypertensive	84.2	84.6	85.0	85.5
Lipid-lowering	66.1	67.9	70.0	71.2

### Association between RC and incidence of AD

During an average follow-up period of 3.69 ± 1.33 years, 312 new cases of Alzheimer’s disease were reported. The number of incident AD cases and the incidence rate for each group are shown in **Table 2**. The incidence of AD in each quartile was as follows: Q1 had 111 cases, Q2 had 55 cases, Q3 had 41 cases, and Q4 had 105 cases. The lowest incidence rate of AD was observed in the Q3 group at 0.0030 per person-year, while the incidence rates were higher in the Q1 and Q4 groups at 0.0075 and 0.0074 per person-year, respectively. After adjusting for age and sex, the relative risk ratios (95% CI) of developing AD for the Q1, Q3, and Q4 groups compared to the Q2 group were 1.878 (1.359-2.594), 0.795 (0.530-1.191), and 1.958 (1.413-2.713), respectively, as shown in **Table 2**. After adjusting for multiple AD-related factors (age, sex, BMI, SBP, DBP, HbA1c, use of lipid-lowering medications, use of antihypertensive medications, use of glucose-lowering medications, and smoking status), the relative risk ratios (95% CI) for the Q1, Q3, and Q4 groups were 1.891 (1.368-2.613), 0.789 (0.526-1.183), and 1.891 (1.363-2.622), respectively. When RC was considered as a continuous variable, each 1 mmol/L increase in RC level was associated with a 3.47-fold increase in the risk of developing AD (HR=4.474, 95% CI 2.330-8.592).

**Table 2:** Hazard ratios of Alzheimer’s Disease by different levels of residual cholesterol among patients.

	Baseline residual cholesterol (mmol/L)				As a continuous variable
	Quartile 1	Quartile 2	Quartile 3	Quartile 4	
Incident Alzheimer					
No. of participants	4006	3875	3659	3824	
No. of cases	111	55	41	105	
Person-years	14707	14243	13612	14112	
Age- and sex-adjusted HR	1.878 (1.359-2.594)	1.00	0.795 (0.530-1.191)	1.958 (1.413-2.713)	4.881 (2.549-9.346)
Multivariable adjusted HR	1.891 (1.368-2.613)	1.00	0.789 (0.526-1.183)	1.891(1.363-2.622)	4.474 (2.330-8.592)

Multivariable adjusted models included age, sex, BMI, SBP, DBP, HbA1c, use of lipid-lowering medications, use of antihypertensive medications, use of glucose-lowering medications, and smoking status.

**Figure 1:**
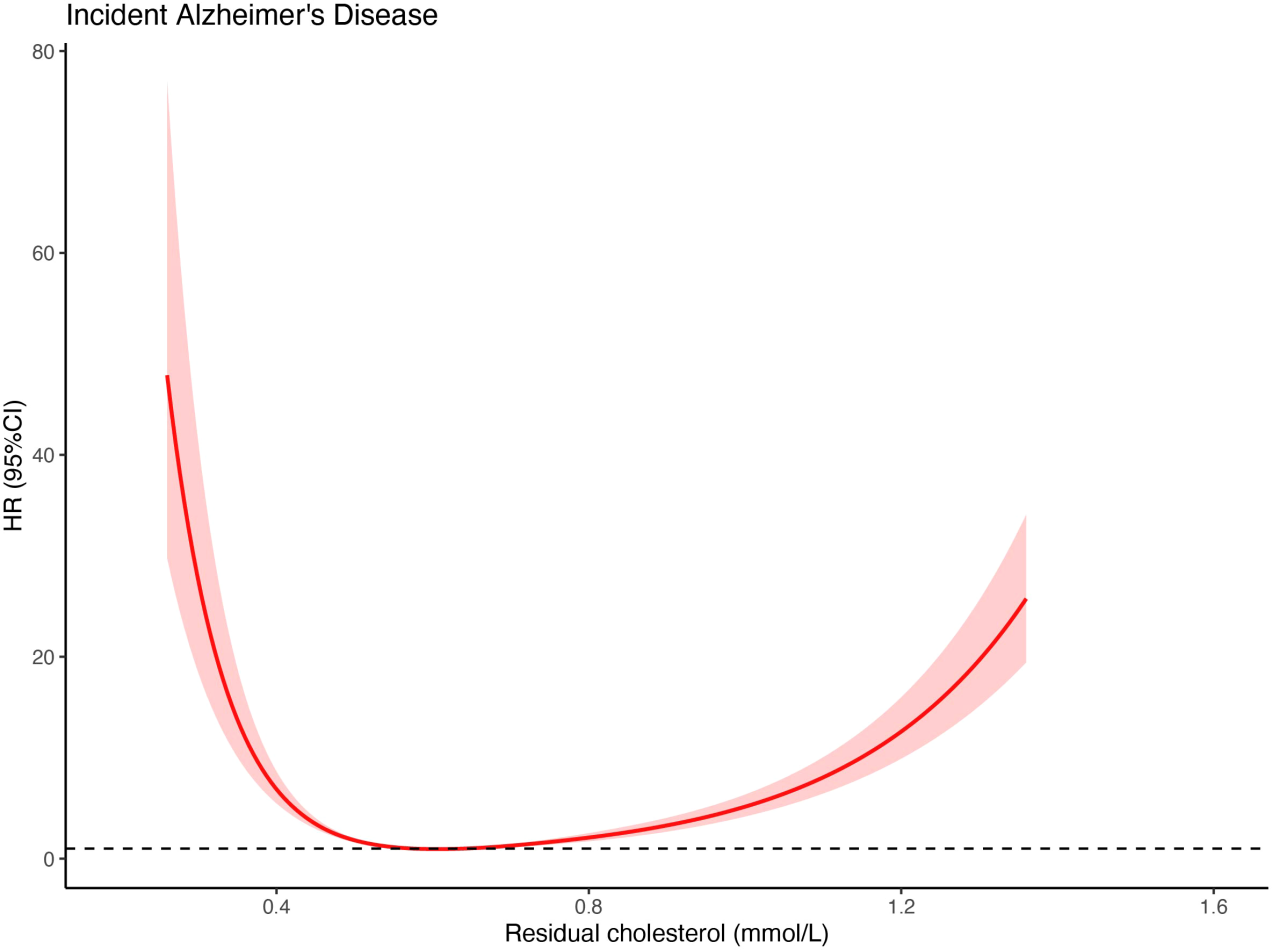
Restricted cubic spline curves for RC and AD. A U-shaped association of RC as a continuous variable with the risk of AD was observed when the restricted cubic spline curve was applied. The lowest hazards of AD occurred when serum residual cholesterol levels were considered as 0.58 mmol/L and 0.64 mmol/L.

As shown in **Figure 1**, the restricted cubic spline plot indicated a U-shaped dose-response relationship between RC levels and the hazard ratio (HR) for developing AD. The lowest risk of AD was observed when RC levels were between 0.58-0.64 mmol/L (P for non-linearity < 0.01). The risk of AD increased as RC levels decreased below 0.58 mmol/L and as RC levels increased above 0.64 mmol/L. This result is consistent with the trend observed in the risk analysis based on RC quartiles (**Table 2**).

### Subgroup analyses

Due to significant differences in gender distribution, HbA1c levels, and the proportion of patients using lipid-lowering medications, antihypertensive medications, and glucose-lowering medications among the four groups, we conducted subgroup analyses based on these indicators. The trend between RC levels and the risk of AD incidence remained consistent across the subgroup analyses. The U-shaped correlation trend between baseline RC levels and the risk of AD incidence was consistent regardless of gender, HbA1c levels (<6.5% or ≥6.5%), use or non-use of glucose-lowering medications, use or non-use of antihypertensive medications, and use or non-use of lipid-lowering medications (see **Supplementary Table**).

### Informed consent

We did not obtain informed consent from individual participants involved in our study because we used anonymized data compiled from EHRs.

## Discussion

In this study based on the real-world data, we found that baseline serum RC levels were significantly associated with the risk of AD. Participants with baseline RC levels between the 25th and 75th percentiles had relatively lower risks of AD, whereas those in the 0-25th and 75-100th percentiles had approximately twice the risk of AD compared to the middle groups. This trend persisted even after adjusting for age, sex, weight, blood pressure, HbA1c, medication use, and smoking status. Subgroup analyses based on different baseline characteristics showed that the previous results were consistent across different subgroups. The relationship between serum RC levels and the risk of AD followed a U-shaped dose-response pattern, with the lowest risk observed when RC levels were between 0.58-0.64 mmol/L. Both higher and lower RC levels increased the risk of AD.

To our knowledge, few studies have investigated the correlation between RC levels and AD. A cross-sectional study by Yating Ai et al. found that high RC levels were an independent predictor of amnestic mild cognitive impairment, particularly manifesting as naming impairment in the elderly. The researchers suggested that the predictive role of RC should be confirmed in longitudinal studies (21). Additionally, Michelle M Dunk et al. used Mendelian randomization to find that high RC levels were associated with an increased risk of dementia and cognitive impairment (9).

Extensive epidemiological data support the correlation between RC and cardiovascular diseases. Large cohort studies in community populations and those with type 2 diabetes have confirmed the causal relationship between RC levels and cardiovascular disease risk, independent of traditional cardiovascular risk factors such as LDL-C and Apo-B (22, 23). Therefore, some suggest that RC levels should be considered a novel lipid-lowering treatment target to reduce cardiovascular events (18). Additionally, RC can independently predict the occurrence of non-alcoholic fatty liver disease (24) and long-term mortality in patients with metabolic dysfunction-associated fatty liver disease (25). There is also a positive correlation between residual cholesterol concentration and the occurrence of depression (26).

RC consists of partially lipolyzed, triacylglycerol-rich lipoproteins, including cholesterol content from very low-density lipoproteins and IDL in the fasting state, as well as cholesterol from chylomicron remnants in the non-fasting state (27). Currently, no basic research has proven that RC directly participates in the pathogenesis of AD.

However, cholesterol homeostasis is known to be closely related to AD. Targeted deletion of astrocyte cholesterol synthesis significantly reduces amyloid and tau burden in mouse model of AD (7). Low level of cholesterol in neurons inhibits Aβ accumulation and enables the astrocyte regulation of Aβ accumulation by cholesterol signaling (28). Cholesterol homeostasis is impaired in Alzheimer’s disease (29).

A study by Montse Guardiola et al. identified common genetic variations between AD and metabolic syndrome, finding five SNPs significantly associated with lower cholesterol transport levels in remnant lipoprotein particles (30). Apolipoprotein E (apoE) epsilon variants and subtypes are also associated with dementia (31). ApoE can bind to low-density lipoprotein receptors and chylomicron remnant receptors (32). Based on published studies, we hypothesize that the correlation between RC and AD incidence might be related to certain apoE subtypes transporting cholesterol into brain tissue. However, this is only a hypothesis and requires more direct evidence. Controlling RC levels within a moderate range might be helpful to reduce AD risk, but this speculation needs further prospective research.

The main strengths of this study are the large sample size. Despite the relatively low incidence of AD, the large sample size reduced possible statistical errors and provided reliable statistical results. Additionally, standardized biochemical testing and standardized medical history questionnaires provided reliable data sources, ensuring comparability between different groups. Furthermore, the average participant age of over 70 years is within the common age range for AD onset, and the average follow-up period of more than three years helps identify potential AD cases, enhancing the reliability of the results. Of course, as the follow-up period extends, we expect to obtain more data related to AD incidence in subsequent follow-ups to aid further analysis.

However, some limitations of this study should be noted. First, the study population is limited to the one center with Chinese population, which may introduce bias in the source of subjects. Therefore, the conclusions should be cautiously interpretated, as AD incidence characteristics and lipid metabolism may vary across different populations. Secondly, the current study is observational, which is unable to interpret a causal relationship between RC and AD. Additionally, due to data collection limitations, some AD-related risk factors were not brought in to analysis, such as diet, physical activity, and socioeconomic status. Therefore, it is necessary to include a broader range of influencing factors in other populations to verify this conclusion.

## Conclusion

In summary, RC levels have a U-shaped correlation with the risk of AD, with both low and high RC levels indicating a higher risk of AD. RC levels may be considered as a potential biomarker for AD incidence. The underlying mechanisms of this phenomenon require further exploration.

## Data Availability

The original contributions presented in the study are included in the article/**Supplementary Material**. Further inquiries can be directed to the corresponding author.
