# Supplementary material for: Association between calculated remnant cholesterol levels and incident risks of Alzheimer’s disease among elderly patients with type 2 diabetes: a real-world study
Source: Front Endocrinol (Lausanne). 2024 Nov 29;15:1505234. doi: 10.3389/fendo.2024.1505234 (PMC11637845; doi:10.3389/fendo.2024.1505234)
Supplement: Supplementary file 1 [file Table1.docx]

Supplementary Table. Subgroup analysis according to different baseline characteristics in association of RC with incident Alzheimer’s Disease

|  | Residual cholesterol (mmol/L) | | | | As a continuous variable |
| --- | --- | --- | --- | --- | --- |
|  | Quartile 1 | Quartile 2 | Quartile 3 | Quartile 4 |  |
| Sex |  |  |  |  |  |
| Male | 2.409 (1.354-4.288) | 1.00 | 0.788 (0.373-1.667) | 2.278 (1.263-4.110) | 3.118 (1.029-9.446) |
| Female | 1.679 (1.133-2.488) | 1.00 | 0.774 (0.478-1.253) | 1.722 (1.161-2.554) | 5.332 (2.378-11.955) |
| HbA1c, % |  |  |  |  |  |
| < 6.5 | 1.917 (1.172-3.135) | 1.00 | 0.667 (0.345-1.293) | 1.414 (0.828-2.415) | 0.890 (0.273-2.906) |
| ≥ 6.5 | 1.736 (1.137-2.650) | 1.00 | 0.919 (0.560-1.57) | 2.136 (1.411-3.234) | 9.711 (4.641-20.322) |
| Lipid-lowering medications |  |  |  |  |  |
| No use | 2.868 (1.451-5.668) | 1.00 | 1.263 (0.555-2.876) | 2.834 (1.399-5.739) | 1.756 (0.483-6.388) |
| Use | 1.809 (1.252-2.614) | 1.00 | 0.758 (0.478-1.203) | 1.835 (1.267-2.658) | 6.583 (3.124-13.870) |
| Antihypertensive medications |  |  |  |  |  |
| No use | 3.039 (0.996-9.280) | 1.00 | 1.200 (0.298-4.840) | 3.656 (1.180-11.331) | 3.896 (0.582-26.061) |
| Use | 1.771 (1.261-2.488) | 1.00 | 0.749 (.495-1.133) | 1.787 (1.266-2.523) | 4.509 (2.256-9.012) |
| Glucose-lowering medications |  |  |  |  |  |
| No use | 3.482 (1.782-6.807) | 1.00 | 1.077 (0.466-2.489) | 1.548 (0.715-3.351) | 0.090 (0.019-0.433) |
| Use | 1.566 (1.074-2.284) | 1.00 | 0.832 (0.530-1.306) | 2.007 (1.390-2.899) | 11.611 (5.929-22.739) |

Data are hazard ratios (95% confidence intervals). Multivariable adjusted models included age, sex, BMI, systolic blood pressure, diastolic blood pressure, HbA1c, use of lipid-lowering medications, use of antihypertensive medications, use of glucose-lowering medications, and smoking status
